# Inpatient Institutional Care: The Forced Social Environment

**DOI:** 10.3389/fpsyg.2021.690384

**Published:** 2021-10-07

**Authors:** Emma C. Joyes, Melanie Jordan, Gary Winship, Paul Crawford

**Affiliations:** ^1^Population Health Sciences Institute, Faculty of Medical Sciences, Newcastle University, Newcastle Upon Tyne, United Kingdom; ^2^Law and Social Sciences, Faculty of Social Sciences, University of Nottingham, Nottingham, United Kingdom; ^3^School of Education, Faculty of Social Sciences, University of Nottingham, Nottingham, United Kingdom; ^4^Institute of Mental Health, Faculty of Medicine & Health Sciences, University of Nottingham, Nottingham, United Kingdom

**Keywords:** recovery, social connectedness, interpersonal wellbeing, forensic mental health, inpatient secure care, ethnography, holistic wellbeing, therapeutic communities

## Abstract

The landscape of mental health recovery is changing; there have been calls for a shift from the clinical expertise being the dominant voice within mental healthcare towards a more personalised and collaborative service that supports those in need of mental healthcare to define what recovery is for the individual. Within this new recovery movement, there has been a recognition of the importance of the social environment in which individuals are situated and the relationship of this to mental health and wellbeing. Included in this is the importance of an individual’s role within society and the ways in which knowledge, such as experts by experience, can hold an important value. The argument then, is that social connectedness forms part of the recovery journey and that relationships can help us develop or re-connect with who we are in powerful ways. Such a view has only been strengthened by the recent and ongoing global COVID-19 pandemic. Within the UK, discussions of the importance of our wellbeing have become commonplace within the context of restricted social contact. With this heightened awareness of how the social contributes to wellbeing, it is important to consider the environments in which those in receipt of mental healthcare are situated. One of which is institutionalised care, where it is commonplace to restrict social contact. For example, by virtue of being within a locked environment, individuals’ freedom of movement is often non-existent and thus contacts with those not residing or working within the institution is restricted. While such restrictions may be deemed necessary to protect the individual’s mental health, such environments can be unintentionally toxic. Data are presented from an ethnography that was conducted within an inpatient forensic mental health hospital in the UK to highlight the problematic social environment which some individuals experience. Key interpersonal issues are presented, such as, trust, racism, the threat of physical violence and bullying that was experienced by staff and residents at the hospital. Consideration is given to the coping strategies enacted by residents and the pathologising of such behaviour. The consequences on interpersonal wellbeing are explored.

## Introduction

There has been a growing recognition of the importance of holistic wellbeing, particularly within the context of the global pandemic which has led to the restriction of social contacts. Public health measures resulting in the restriction of social movement increased worldwide as the spread of COVID-19 infection continued (Han et al., 2020). Isolation, quarantine and social distancing are all measures which are often utilised to reduce infection rates and thus the spread of a respiratory virus (Wilder-Smith and Freedman, 2020). For the United Kingdom (UK), the first lockdown measures were brought into force in March 2020. Since then, there have been further lockdowns with varying degrees of restrictions nationwide. Discussions relating to the importance of mental health and wellbeing have become commonplace within the context of the pandemic and the associated restrictions on social contact and distancing measures. Holistic wellbeing principles have been the foundation of much of the debate and accompanying research around the global pandemic and the impact upon individual’s mental health and wellbeing. For example, calls for society to maintain their social connectedness began appearing within the literature, albeit through remote means (Usher et al., 2020) and recommendations for the promotion of social connectedness have also been published (Mental Health Foundation, 2020).

While the literature recognising the importance of interpersonal wellbeing is increasing and such debates are becoming central to discussions relating to mental health and wellbeing, it is important to consider the development of such conversations within mental healthcare. Traditional recovery models (i.e. clinical recovery) typically focus on the recovery of the individual through the alleviation of symptoms (Slade, 2009; Winship, 2016). Mental healthcare has been shifting its focus from this dominant model towards a more holistic approach to mental health and wellbeing. These developments, termed by Winship (2016) as ‘new recovery’ models, are underpinned by learning and experiential models associated with recovery colleges and experts by experience. Holistic wellbeing incorporates the move towards ‘an acceptance of the definition of health as being more than the absence of illness…[towards] whole-person approaches to creating and sustaining health’ (Stuckey and Nobel, 2010, p.254). Thus, within mental healthcare, recovery models have begun to recognise the importance of a more holistic view of recovery, one that includes and values social relationships (Jacob, 2015). As a result, recovery frameworks include social connectedness as one of the key elements of recovery (Leamy et al., 2011). Furthermore, social roles and networks are argued to provide an opportunity to find meaning through the development of social identity (Winship and Barker, 2016). It is recognised, however, that personal or indeed new recovery principles can sharply contrast with the clinical recovery model that pervades healthcare professional practice. Such principles can be overshadowed in the clinical context where mental ill-health is viewed as a deficit or a disorder and where the focus is on diagnosis and clinical treatment (Davidson and Roe, 2007; Brown and Manning, 2018). Within inpatient secure care, such principles are particularly challenged due to the restrictive nature of the environment. This paper reports on research within such an environment – the inpatient forensic mental health context – and includes examples from the research that provide an insight into the interpersonal issues experienced by staff and residents. Extant evidence from the custodial literature demonstrates that the relationship between clinicians and mental health patients within secure settings involves added complexity, due to the institutionalised nature of service delivery and receipt – context is crucial (Jordan, 2010, 2011, 2012). This research embraces the existing call for complexity – in both critical analysis and implications for praxis. The next section explores literature on mental healthcare practice in these settings as a prelude to the discussion of the research.

### Forensic Mental Health: Secure Care

Within the inpatient forensic mental health context, individuals are associated with both the label patient and the label offender. Robertson et al. (2011, p.473) explain that:

modern forensic services have their theoretical roots in two quite different paradigms — the treatment of mental illness (a psychopathology paradigm) and the assessment and management of risk (a risk paradigm).

The competing frameworks of care and custody are complex (Hinshelwood, 2001). Individuals being placed at the level of security that matches the risk of the individual is an accepted notion in UK practice (Crichton, 2009). However, debates about appropriate risk levels have been surfacing in the context of recovery and the promotion of autonomy (Tomlin et al., 2018). Service user involvement models can seem antithetical to traditional clinical models of care, which values professional knowledge, and also secure environments whereby restrictions on the self are rife. Safety is highly valued within the psychiatric inpatient environment, and risk management is ‘the cornerstone of nursing care’ (Slemon et al., 2017, p.1). The secure features of such environments can provide psychological safety for individuals in receipt of care (Winship, 2000; Mezey et al., 2010; Stenhouse, 2013), particularly for those who have been subjected to abuse (Adshead, 2004). However, it is important to consider the influence of the social environment on wellbeing and the therapeutic milieu when the aim of residency within such an institution is to provide treatment and recovery and the ‘social skin’ is a key component of secure mental health services (Winship, 2000, p.176). As such, it is argued that ‘[s]ocial inclusion and the availability of supportive relationships are undoubtedly integral features of mental health and well-being’ (Middleton, 2017, p.264). Such a claim can now be seen as capturing the essence of the pains felt by those who can no longer spend time with loved ones as a result of restricted social contact during the COVID-19 pandemic.

Secure residential environments have been found to be unintentionally toxic (Davies, 2004) and may exacerbate problems or hinder recovery (Drennan and Wooldridge, 2014). The deterioration of mental and physical health, social isolation (Davies, 2004) and loss of liberty (Gillard et al., 2012; Norvoll and Pedersen, 2018) are commonly associated with inpatient mental healthcare. The health and wellbeing of the individual, therefore, are reported as being significantly influenced by the restrictive and non-autonomous environment associated with institutional care. However, it is the ‘social skin’ that can be powerful in transforming identities (Winship, 2000, p.176). For example, positive interpersonal interactions contribute towards social connectedness (Baumeister and Leary, 1995) and positive therapeutic relationships can promote wellbeing (McKeown et al., 2016a; Marshall and Adams, 2018). Positive and supportive social environments are particularly important when diminished social networks are found to negatively influence wellbeing (Kawachi and Berkman, 2001; Hare-Duke et al., 2018). These social dimensions of health have been recognised within the Enabling Environments initiative and subsequent standards outlined by the (Royal College of Psychiatrists, (2013). Central to this initiative is an attempt to ‘identify the key features in any setting which foster a sense of connected belonging’ (Johnson and Haigh, 2021, p.1). This paper considers the ‘social skin’ in relation to the community of individuals who live within such environments and also considers the pressured environment that staff endure (Winship, 2000, p.176).

With this increased recognition of the importance of social connectedness to mental health and wellbeing, it is important to consider the influence of restricted contact for those within the secure inpatient setting, where restrictions on social movements are part of the fabric of the institution. However, it is recognised that recovery-oriented practice which promotes patient-centred care can conflict with the dominant care practices which focus on risk and containment (Stickley and Wright, 2011). Incorporating new recovery within current practice in mental healthcare, within an environment that is known to be unintentionally toxic certainly, has its challenges, and this was the focus of the ethnographic research, involving participant observation, carried out by the first author between 2016 and 2017 and reported in this paper.

## An Ethnography: Inpatient Forensic Mental Health Hospital (UK)

Ethical approval was successfully obtained from an NHS Research Ethics Committee, which specialised in qualitative research and the Mental Capacity Act (2005, 16/LO/0471). The research protocol was provided with a favourable opinion.

### The Research Site

The hospital was situated within the United Kingdom and had three low secure wards: two male and one female, which ranged from 15 to 19 beds on each ward. The hospital housed individuals who had a history of offending and had been assessed as requiring care for their mental health. Typically, residents were detained under the Mental Health Act (1983/2007), with varying restrictions relating to their perceived risk to themselves and others. There were various departments which supported the wards, these included administration, maintenance and housekeeping, kitchen, nursing and healthcare, consultants, social work and therapy, including occupational therapy and psychology. The courtyards and building were secure and lined with CCTV cameras. The wards all had a staff room which overlooked the common seating area. The bedrooms were situated along corridors which led to the main ward area. Each staff room had a live feed displayed on a computer which showed the ward. All movement in and out of the hospital was monitored, and access was granted on request.

### Participant Observation

Participant observation is both a methodology and a method – it is an approach to understanding the group under study and a way to collect data through observing participants (Howitt, 2010). Participant observation is argued to facilitate the researcher to become an insider to the culture being studied (Whitehead, 2005). Social intercourse pertaining to everyday conversations has been suggested to be pertinent for the development of trust and is the basis of rapport (Hammersley and Atkinson, 1995). Engagement in everyday conversations is suggested to be the route to facilitating observations, events and meaningful conversations (Davies, 2008). Overt participant observation was adopted as a methodology and a method within this study in order to understand daily life at the hospital. The first author spent over 300h within an inpatient forensic mental hospital in the UK, observing and participating within daily life on the three wards. A total of 14 staff and 14 residents participated in the observational element of the research. It should be noted that individuals within forensic mental health services are referred to as offenders, patients, service users and mentally disordered offenders (Prins, 2010). A multitude of professions now work within the field (Rogers and Soothill, 2008) therefore, these terms are used interchangeably within and across forensic mental health services and the criminal justice system. However, within this paper, the use of the terms resident or patient does not imply overlooking the individuality of those in receipt of care, but simply to identify individuals as living in such services or receiving care. The term patient is used when discussing individuals who are in receipt of care from forensic mental health/mental health services more widely and resident is used when referring specifically to the research site and those that were residing at the hospital at the time of the research. In fact, the view of this paper is that the use of labels (e.g. mentally disordered offenders) elicits a negative ethos for those who live with these and for those who utilise them (Dixon, 2015).

The participant observer role would often fluctuate from complete observer, where the ‘researcher does not interact with people’ (Bryman, 2004, p.301) to participant-as-observer, where the researcher participates in the daily lives of the group being studied which involves regular interaction. The roles are suggested to be related to the degree of acceptance of the researcher in the social group (Davies, 2008). This developed over time through the building of relationships with staff and residents influencing the participation of researcher within everyday encounters. However, this would also fluctuate depending on the type of activity that the researcher was undertaking. For example, author one undertook a shadowing role where they shadowed the maintenance team, reception, domestic staff, kitchen staff, the psychology department and the occupational therapy team. The researcher was present at formal clinical meetings, such as ward rounds, where a representative from the nursing, medical, psychology, occupational therapy and psychiatry departments were present. During these times, the researcher was more aligned with the complete observer role. However, the researcher also continued to spend time on the wards, with different staff, such as doctors, healthcare assistants and nursing staff. The researcher ate lunch with residents on the ward and also with non-ward staff and thus spent time in the staff dining area, sometimes observing and sometimes engaging with staff, for example, spending time at lunch with the hospital and clinical management members of staff. The researcher also undertook the role as a staff helper as a way to legitimately participate within the community. The staff helper role, along with the adoption of a staff dress code, assisted with creating a ‘front’ so that access to the participant observer role could be gained (Bryman, 2004, p.299). Moreover, this role assisted in building rapport with various staff and residents and also assisted with undertaking more of a participant-as-observer role.

Within this paper, a data vignette is presented. A vignette ‘is a vivid portrayal of the conduct of an event of everyday life’ (Erickson, 1986, p.149). The intention of a vignette is to elicit ‘emotional identification and understanding’ (Denzin, 2001, p.141) and thus, such an approach facilitates ‘bring[ing] research to life’ (Ellis, 1997, p.4). This approach has also been suggested to allow the reader ‘to sense some of the evocative power, embodiment, and understanding of life that comes through the concrete details of narrative’ (Ellis, 1997, p.9). Such moments, while representative of typical moments within the field study site, also serve as a series of informative critical incidents for the researcher in which key features of community life were salient.

### Interviews

Unstructured ethnographic interviews were conducted during fieldwork – these are informal conversations where the researcher asks questions in order to gain further insight in to an area of interest (Whitehead, 2005) and represent a natural conversation (Kelly, 2010). Semi-structured one-to-one interviews were conducted later during fieldwork once relationships had been developed in the field (Madden, 2010). The interviews took place between month four and nine of the research. One of the interviews was conducted earlier than expected due to one of the participants moving on from the hospital during month four. In total, 11 interviews were conducted with two occupational therapy staff, one education support worker (two female and one male) and seven residents (two female and six male).

The interviews were audio-recorded and ranged from 2min to 1h 40min. The broad range in time reflects the issues encountered when conducting interviews within the busy ward environment and the timing of the interview in relation to institutional routines. For example, one of the interviews was interrupted because the General Practitioner had attended the ward and the resident had requested an appointment. Only one of the interviews was undertaken with no interruptions from institutional regimes. The interviews were transcribed verbatim. The fieldnotes also contained data related to the interviews, which included initial insights which served as theoretical codes and methodological insights, such as body language which revealed insights into what was said during the interview, which would not be captured *via* audio recordings. The behavioural descriptions inform some of the results presented within this paper.

### Data Analysis

The data collection and analysis stage of ethnography are often intertwined, and these are not distinct phases of the research process (Coffey, 2018). Initial theoretical codes were developed during the fieldwork, particularly during the writing of fieldnotes as this process often ‘heightens and focuses… [the] interpretive and analytic process’ (Emerson et al., 1995, p.100).

The constructionist ontological position is adopted, and the premise of this research is, therefore, situated in the view that social phenomena are constructed (Bryman, 2004). An emic and etic approach to knowledge was adopted. For example, the emic approach to knowledge is adopted in order to understand the local interpretation (Berry, 1999) of community life at the hospital. Thus, an approach to understanding ‘components of a cultural system from the perspective of the group being studied’ is considered (Whitehead, 2004, p.16). An inductive approach to knowledge, therefore, underpins this research, in that the aim is to understand the perspectives of those who work and reside at the hospital. An etic approach considers the researcher’s theoretical ideas (Hammersley and Atkinson, 1995). Such an approach is adopted iteratively, and the application of theory emerges over time alongside data collection (Goldbart and Hustler, 2005).

## Results

This paper presents salient findings from the research. Data are presented from three themes, and these are (i) Trust, (ii) Racism and the Threat of Physical Violence and (iii) Avoidance Rituals. This paper focuses on the interpersonal issues experienced by staff and residents within the inpatient forensic mental health context. During the study, it was observed and also discussed by those within the institution that individuals (both staff and residents) experienced racial abuse, threats of violence, intimidating behaviour and trust issues, which sometimes led to avoidance behaviour. Avoidance behaviour within the inpatient forensic mental health context can be challenging when ward routines force individuals into communal spaces. These results are presented below from observations in the form of a data vignette, fieldnotes and data from one-to-one interviews with individuals who were receiving care at the hospital where the research was conducted. Throughout the results section, the first person is used and is from the perspective of the first author who conducted the ethnographic research. Such a convention is typical within ethnography (Gullion, 2016) as the etic and emic understandings of the culture under study are viewed through the lens of the researcher. Pseudonyms have been used throughout this paper.

### Trust

During the interviews with the residents, I was informed of the issues relating to trust that some of the residents experienced. Ryan, a resident at the hospital, reported that this was an issue for a few of the individuals on the ward, and it was explained that:

‘… everyone talks to everyone, erm not everyone’s trusted on here. There are a couple of people that are hard to trust. But currently [we don’t] exclude anyone from anything because even if we dislike them, we still tolerate them, and we still do groups with them’.

*[Resident interview: Ryan]*

Ryan explains that fellow residents are not excluded from the group, and everyone is tolerated to a certain degree. However, there are trust issues among the community of residents on the ward. Ryan indicates that some individuals are tolerated; however, tolerance does not describe a mutual or reciprocal relationship. Tolerance is something quite different to social connectedness. Furthermore, Ryan also uses the term: ‘we’ and not ‘I’ when describing inclusion within the community. It may be that Ryan identified within an in-group of individuals on the ward, such as a group of residents or indeed the occupational therapists who ran much of the therapeutic groups. However, this phrase was not questioned at the time of the interview.

During an interview with Ian, another resident at the hospital, it was explained that he had experienced an incident with a fellow resident and this incident had led him to lack trust in other residents, he shared:

‘Well at first after doing that, I felt cautious of other in-mates, well other patients, and it took me, after that, two years to trust people again’.

*[Resident interview: Ian]*

While Ian reports that it took 2years to trust people again, he also explained how he does not trust fellow residents on the ward to make a drink for him:

‘I never touch drinks off wards even if a patient comes up to me and says: ‘Do you want coffee?’ I say: ‘No’. I’m quite wary of things like they’re quite far [away at the] end [of the ward] and you don’t know what they’re doing. So, I don’t take drinks off people’.

*[Resident interview: Ian]*

Ian explains how past experiences on the ward has led him to not trust others that are part of the ward community. These are individuals that Ian had spent considerable time with, but felt distrustful of all the other residents. This was the community in which Ian lived and spent considerable time with.

### Racism and the Threat of Physical Violence

Prior to commencing the research, I attended the hospital for a meeting with the hospital manager. We discussed the research and the interesting insights that could be gained from conducting the research at this particular site. The meeting also focused on the physical and verbal abuse risks that I would be undertaking when I spent time on site. I was told that a student had not been physically hit before, but that there was always a chance this could happen. This did not come as a surprise to me and I could understand why this was being made clear to me before I began the research. However, the risk of physical harm loomed over me on every visit.

*[Fieldnotes]*

The following excerpt is a data vignette which showcases a moment of tension which is representative of commonly occurring situation at the hospital.

A group of residents had returned to the ward after a visit to the community. The Occupational Therapy team had been thanking everyone for a lovely afternoon out in the community. Shortly after this, the majority of the residents had dispersed to their rooms or had gone outside for their last cigarette of the day.

I headed back out onto the open ward area, just as a commotion developed between a resident and two staff members. I heard: ‘Fuck off you [racial slur] bastard!’. I was alone at the other end of the ward. I looked over and the resident was sat down and glaring at the staff. I decided to head back to the safety of the staff base, where I found myself alone for a moment. Three members of staff then burst into the staff base; the more senior member of staff remarked in a stressed tone: ‘Oh my God!’. She looked at me and apologised and then immediately looked back at the staff, swearing: ‘Oh my God, what the fuck!’. She explained that the resident had a cup of boiling water [which was being threatened as a weapon] while another staff member added that he also put sugar in it. They rushed back out onto the ward leaving me alone again in the staff base. Another member of staff appeared, visibly angry, glaring at me. I could see that he was thinking through his next move. Another staff member followed him in and advised him to leave it to the other staff, it wasn’t necessary for him to get involved. He refused and went back out to the ward and over to the scene of the commotion.

Kerry appeared in the staff base and stated: ‘Shall we get you off the ward?’. I hesitated and she asked again, and this time I answered: ‘Yes, okay, thank you’. She led me out onto the ward, cautiously positioning herself in a protective manner to guard me from the commotion taking place. She pressed the green door release and sent me through saying: ‘Thanks, Emma’.

I reflected at the time: Whilst I had been informed of the likelihood of aggressive and violent acts at the hospital, this moment felt incredibly stressful and threatening. The intensity of the situation was built upon by the reaction of the staff that I knew were typically calm, poised, and professional. However, the situation had evoked strong emotions for all involved.

*[Vignette]*

Racial issues were also apparent between residents at the hospital. Imran explained that he would watch TV in his room in order to avoid certain residents because of the abuse he had previously encountered:

‘it depends ‘cause like if it's quite busy or if it's, or if erm, depending on which erm patients, which patients are on the ward, erm because there are some of them that I avoid, I've had erm racial abuse off some of the patients, so I avoid them purposefully’.

*[Resident interview: Imran]*

Imran responded to the group dynamics of the social environment on communal areas of the ward by observing who was present within these spaces and responded accordingly. If the individual(s) who had verbally attacked Imran were present, he retreated to his room. Avoidance tactics undertaken by residents at the hospital is further explored in the next theme: avoidance rituals.

### Avoidance Rituals

Within the previous section of the results, Imran explained how he ‘avoid[ed]’ those patients who’d been racially abusive towards him ‘purposefully’. Imran also further explained how he avoided residents who he had interpersonal issues with and describes that he is irritated by the resident’s mannerisms. The interview excerpt below highlights Imran’s feelings towards Adam, including his treatment by fellow residents.

‘I don’t do as much [Occupational Therapy] OT as I used to, and that’s to avoid Adam and he does a lot of OT, he does practically everything…so I avoid him, not because I don’t like him, I think he’s alright, but I, I don’t have it in me, to put up with him and his mannerisms, for a whole week and like not even a little bit… So yeah, so I have snapped at him a few times and other people as well, but for different reasons’.

*[Resident interview: Imran]*

I reflected at the time: Adam, on the other hand, actively engaged in the occupational therapy timetable. Adam established close working relationships with the staff, particularly the occupational therapy team who were friendly towards him and he would often make them laugh. Adam enjoyed spending time with staff and thus participated in all the occupational therapy groups available. However, his presence led Imran to undertake avoidance behaviour as interactions with him were considered unfavourable. I have often observed that Adam was rejected by the residents on the ward.

*[Fieldnotes]*

The excerpt below highlights an example of this bullying behaviour from James towards Adam, who were both residents on one of the male wards.

During a music session with Charlie on the ward, four residents and Charlie had taken part in a group music making session. Adam was given the task to sing; he was great at making up lyrics on the spot. The session had ended, and we headed back out to the ward. He began to sing on the ward. James suddenly became irritated and said: ‘Shut up Adam!’. Adam continued to sing. James continued to shout at Adam: ‘Shut the fuck up Adam!’. Eventually Adam stopped and walked off to his room.

*[Fieldnotes]*

During the interview with one of the residents on the female ward, interpersonal conflicts also became apparent. During our conversation about the community on the ward, Charlotte, was hesitant to answer, and her eyes were fixed on a fellow resident who could be viewed through the window of the interview room; the resident was shouting. Charlotte then explained:

“I try to spend as much of my time either doing activities or in my room and staff doesn’t like it so much, being in my room, but I’m like ‘I don’t want to sit out there on the ward’. And they're like, ‘Why you isolating yourself?’ And it’s like ‘You try living on here, every week, and you would isolate yourself in your room’”.

*[Resident interview: Charlotte]*

Charlotte also engaged in avoidance rituals by participating in activities or spending time in her room, in order to steer clear of particular individuals on the ward. This was despite staff not encouraging such behaviour.

Residents were found to adopt avoidance rituals in order to protect themselves from the upset caused by fellow residents, which influenced participation within community activities and which in turn also minimised opportunities for social interaction and the potential for social connectedness. However, staff challenged residents when they retreated to their individual bedrooms.

## Discussion

The themes highlighted within this paper illuminate the challenging environment that the staff work within and that the residents experience as part of community life. The dual role of carer and custodian for staff creates tensions in their role (Kurtz, 2002; Jacob et al., 2008), and the emotionally challenging environment is argued to influence the high burnout within forensic mental health staff (Nathan et al., 2007; Johnson et al., 2018). Caring for individuals that society chronically marginalises can lead to complex emotions, such as feelings of disgust, repulsion and fear; however, staff are often not provided the supportive reflective space required to explore these intense feelings (Jacob et al., 2008). It is recognised that the staff-patient relationship can be challenging for both staff and patients (Adshead, 2004; Aiyegbusi, 2009b) but the introduction and maintenance of positive relationships within care hold an important therapeutic value (Middleton, 2017; Marshall and Adams, 2018). Clinical supervision is crucial for managing relationships to promote a therapeutic environment (Aiyegbusi, 2009b). Moreover, it has been noted that those employed with non-clinical staff roles would benefit greatly from clinical supervision. For example, prison officers provided with reflective spaces has led to psychological informed practice, improving encounters between prison staff and those detained within the prison system (Winship et al., 2019). This paper has highlighted a moment in which staff members were visibly distressed by the racial abuse that was being directed towards a staff member. While there was clear outrage being expressed, this was contained to the confines of the staff room, and thus, the emotional outbursts enacted by staff were visible only between staff. Nonetheless, the experience was visibly upsetting for the staff and the moment was certainly challenging. It has been reported that nursing staff are expected to cope by hiding emotion when faced with aggression; however, such experiences can be devasting, which ‘has the potential to create and sustain negative emotions’ (Deans, 2004, p.35), which would inevitably influence the therapeutic milieu.

Racial abuse was observed and discussed within the one-to-one interviews, which was experienced by both staff and residents at the hospital. This finding is supported by research which found verbal racial abuse was directed at both staff and other patients (Stewart and Bowers, 2013). Verbal racial abuse can also be described as interpersonal racism, which has been argued to be inter-related to structural racism in that these everyday social encounters represent the discrimination of Black, Asian, and ethnic minorities that permeate society (Younis, 2021). The over-representation of individuals of Black ethnicities is prevalent in mental healthcare (Browne, 2009; Care Quality Commission, 2011). Furthermore, individuals from Black and minority ethnic communities are disproportionally detained under the Mental Health Act (Singh et al., 2007). The link between race and mental ill-health has, unsurprisingly, historical roots. For example, approaches to knowledge gain regarded as objective or indeed ‘scientific’ have historically been underpinned by biased white supremacist ideals thought to evidence differences in race (Rogers and Pilgrim, 2014). The pervading nature of racism within society means that the underlying issues are, of course, complex. For example, it has also been argued that structural conditions of disadvantage (i.e. social and economic disadvantage) and racism create an environment in which mental illness becomes more likely (Nazroo et al., 2021). These important debates relating to racism and mental health have been documented and explored more thoroughly elsewhere (see: Fernando, 2000; Rogers and Pilgrim, 2014; Nazroo et al., 2021; Younis, 2021, to name a few). It is worth noting that whilst debates on racial issues relating to mental health are not particularly new, these known while disparities in mental health have remained consistent over the last 60years (Nazroo et al., 2021).

Debates exist on where appropriate interventions should be located to address issues related to racism and mental health. However, it is argued that claims by the Department of Health that ‘the solutions lie in the hands of individuals not institutions’ pass upon the understanding of how institutional racism lays at the intersection (the meso) of the structural and the individual [as cited in McKenzie and Bhui (2007), p.368]. It is argued then that such claims miss the inter-related nature of structural and interpersonal racism by scapegoating to the individual as an anomaly, thus claiming that the individual is unrepresentative of the institution or its practices. If this viewpoint is adopted, the ways to evade such anomalies in thinking is through diversity training which has been noted to be an oversimplistic panacea to racism (Younis, 2021). The static nature of these issues within mental healthcare indicates a pervasive problem in which blaming individuals has been argued to be unhelpful (McKenzie and Bhui, 2007). Within forensic mental health, individuals of a Black and minority ethnic background are ‘increasingly disenfranchised’ due to the further exclusions that an offending history and mental health issues bring (Hui, 2017, p.27). Thus, it is important to consider the racism evident within psychiatric practice (Fernando, 2000) and the implications to the wellbeing of individuals who are on the receiving end of deliberate and direct interpersonal racism that commonly occurs within the institutional community in which avoidance can only be short lived.

This paper highlights the interpersonal issues that were experienced by individuals who were in receipt of care at the hospital, and the acute ward has been described as ‘an especially volatile and unpredictable place in which to live’ (Quirk et al., 2004, p.2581). Interpersonal issues, such as trust, were found within this research, which has also been reported in research undertaken within the forensic and mental health environment (Gilburt et al., 2008; McKeown et al., 2016a). The trauma experienced by individuals on a forensic unit can be intensely painful (Aiyegbusi, 2009a), and forming new attachments is fraught with difficulties (Adshead, 2004). Patients have also described the inpatient mental health environment as a culture of violence, including the violence exhibited by staff through restraint (Gillard et al., 2012) and patients through violent attacks (Gillard et al., 2012; Stenhouse, 2013). It has been noted within the literature that the process of building trust is often problematic for the forensic patient and is commonly played out through psychological or physical attacks; such behaviour is argued to be underpinned by past neglect, and abuse and childhood disruptions are re-enacted within the staff-patient relationship (Mann et al., 2014). However, the perceptions relating to the causes of violence in psychiatric care have been found to differ between patients and staff. For example, staff perceived that a patient’s violence was a symptom of their illness and suggested an increase in interventions, such as, medication and restraint, whereas patients advocated for increased communication from staff and suggested that environmental, interpersonal and their illness were often inter-related (Ilkiw-Lavalle and Grenyer, 2003). Thus, indicating a more complex relationship underpins violence within the forensic environment. A more nuanced account of violence in healthcare has been advocated. For example, Holmes et al. (2012) suggest that the inevitability of violence within forensic care ignores the complexities of institutional contributions and the presence of horizontal violence among staff. Other negative experiences for the forensic patient include feelings of fear and embarrassment (Bonner et al., 2002), and fellow patients report feeling upset when other patients are in distress (Thibeault et al., 2010). Thus, the therapeutic milieu can be interrupted or influenced by the presence of disturbance from other patients, which is a common feature of secure psychiatric care.

Within this research, avoidance behaviour was observed. Adam walked away after being bullied by another individual on the ward. Charlotte tried to avoid other individuals on the ward and retreated to her room. Clarke and Waring (2018) also found that individuals in receipt of inpatient mental healthcare were sometimes unsupportive of each other, which led to isolation and alienation. Research has found that individuals within the inpatient environment utilise avoidance techniques to stay safe (Quirk and Lelliott, 2004) and sometimes adopt a performative role in order to avoid coercive treatment (Gillard et al., 2012). Furthermore, Imran reported that he had stopped engaging in occupational therapy in a bid to avoid Adam, so a performative role was not adopted within this instance; however, active avoidance of Adam led to a lack of engagement with therapy. Research has found that patients can be judgemental of others due to their challenging behaviour (Woods and Springham, 2011), and this was the case for Adam who was at the centre of much conflict.

Residents at the hospital were required to be present in the communal ward area during certain periods of the day, and individuals were actively encouraged not to spend too much time in their rooms. The private space of a bedroom can be viewed as a retreat offering safety, which has been linked to feelings of control (if this can be accessed freely); however, independent time spent in a bedroom is often interrupted by surveillance practices, such as staff visits to monitor activity (Brown and Reavey, 2019). For the case of Charlotte, her behaviour was pathologised as her withdrawing and isolating herself when staff asked her ‘why you isolating yourself?’. Charlotte was adopting avoidance techniques in order to protect herself from interpersonal conflicts on the ward. A lack of engagement within therapeutic activities has been reported to be due to safety concerns, such as feeling unsafe to exhibit expression or lacking trust (Kennedy and Fortune, 2014). The avoidance of social spaces, however, does limit opportunities for social interaction. Thus, institutional regimes that promote individuals to spend time with one another provides an opportunity for interpersonal relationships to form or develop, even if the social situation presents challenges.

Individuals who are situated within the inpatient forensic mental health context may often find the ward environment challenging (Bonner et al., 2002; Quirk and Lelliott, 2004; Thibeault et al., 2010; Gillard et al., 2012; Stenhouse, 2013), but it is important to consider how such feelings may manifest themself in resultant behaviour. Social relationships which are underpinned by distrust or negative social experiences can lead to avoidance behaviour due to protective or safety concerns (Quirk and Lelliott, 2004). However, avoidance of social situations within the ward community also minimises opportunities for social interactions, connectedness and sometimes engagement in timetabled therapeutic activities. Furthermore, it is important to consider the complexities of relationships within the inpatient mental healthcare environment, which are influenced by former negative experiences (Adshead, 2004) and interpersonal issues which continue to present themselves in the highly emotive social environment.

The research was undertaken within a context in which one distinct model of care underpinned practice *–* the dominant clinical model. Alternative models of care exist which place high value on interpersonal relationships, where ‘understanding the institutional dynamics of the social setting is fundamental’ to practice (Campling, 2001, p.365). The therapeutic community model is underpinned by mutuality and cooperation (Winship, 2016), and principles of empowerment, collective responsibility and citizenship are adopted (Stern, 2012). Service user involvement is prominent, and members of the community are involved in the decision-making. Moreover, when community disturbance occurs, these moments are explored and viewed as learning opportunities. The principles of safety and containment are embodied through the community as a support system. As Haigh (2013, p.9) explains: ‘Support systems are important in providing a way in which disturbance is tolerated, distress is held and people are not left isolated and rejected when they are feeling desperate’. Furthermore, the notion that community members have a voice is central, and thus, new recovery, which incorporates personal recovery principles relating to autonomy and citizenship, is provided a platform. Interestingly, therapeutic communities have received much criticism due to the adoption of a treatment model which challenges conventional professional frameworks in that the focus of the community is on the development of social relationships, rather than on professional expertise (Manning, 2010). Importantly, it has been found that everyday social encounters and time outside of structured therapy can play an important role in facilitating change within the therapeutic community setting (Clarke, 2017). Such an understanding of the importance of everyday encounters has been noted and is fundamental to the therapeutic community model (Haigh, 2013; Clarke et al., 2016).

The inpatient mental health environment is complex and fraught with interpersonal difficulties. Forensic environments are further complicated by the duality of care and custody, which bring a unique set of challenges. However, issues raised by this study have implications for mental health practice and wellbeing more generally. In particular, the debates surrounding service user involvement principles and least restrictive practice (Tomlin et al., 2018). It is noted that a myriad of practices is likely to be present within modern mental healthcare practice that represent a hybrid of care which move fluidly between traditional and new recovery models, and such models should be celebrated alongside our heightened awareness of the importance of interpersonal wellbeing. Not least the Enabling Environments initiative indicates the applicability of such principles to various contexts. As noted at the beginning of this paper, the global pandemic has raised awareness of the importance of social connectedness and interpersonal wellbeing. The principles underpinning therapeutic communities are particularly relevant, and collaborative models in which the social environment is central have been successfully implemented within a range of contexts, from schools (MacDonald and Winship, 2016) to prisons (Bennett and Shuker, 2018), and within mental healthcare (Mistral et al., 2002). A first person account promotes ‘[h]ope orientated practice’, which is underpinned by ‘working together collaboratively’. (Chandley and Rouski, 2014, p.87), and while it is recognised that adopting service user involvement principles is complex within secure environments, such principles have been found to be successfully adopted benefitting both service users and staff (McKeown et al., 2016b). However, consideration must be given when implementing therapeutic community principles within the modern healthcare landscape of payment-by-results directives (Gosling, 2016). Nonetheless, the power of supportive relationships which ‘are undoubtedly integral features of mental health and well-being’ (Middleton, 2017, p.264) should continue to be part of the debate as we move towards ‘new meaning and purpose [as society] grows beyond the catastrophic effects of’ the global pandemic (Anthony, 1993, p.527).

## Data Availability Statement

The original contributions presented in the study are included in the article/supplementary material, further inquiries can be directed to the corresponding author.

## Ethics Statement

The studies involving human participants were reviewed and approved by London - Camden & Kings Cross Research Ethics Committee (NHS Research Ethics Committee). The patients/participants provided their written informed consent to participate in this study.

## Author Contributions

EJ conducted the research as part of their doctoral studies at the University of Nottingham. MJ, GW, and PC provided supervision for the doctoral research, contributed and approved the final manuscript. All authors contributed to the article and approved the submitted version.

## Funding

This original research was funded institutionally as part of a larger Arts and Humanities Research Council (AHRC) Connected Communities programme grant (AH/K003364/1), ‘Creative Practice as Mutual Recovery’. The funders had no role in study design, data collection and analysis, decision to publish, or preparation of the manuscript. Open access publication fees are supplied by Newcastle University.

## Conflict of Interest

The authors declare that the research was conducted in the absence of any commercial or financial relationships that could be construed as a potential conflict of interest.

The reviewer TC declared a shared affiliation with several of the authors, MJ, GW, PC, to the handling Editor at time of review.

## Publisher’s Note

All claims expressed in this article are solely those of the authors and do not necessarily represent those of their affiliated organizations, or those of the publisher, the editors and the reviewers. Any product that may be evaluated in this article, or claim that may be made by its manufacturer, is not guaranteed or endorsed by the publisher.
